# A New Flavanone as a Potent Antioxidant Isolated from* Chromolaena odorata* L. Leaves

**DOI:** 10.1155/2019/1453612

**Published:** 2019-06-18

**Authors:** Devi Anggraini Putri, Sri Fatmawati

**Affiliations:** Laboratory of Natural Products and Synthetic Chemistry, Department of Chemistry, Faculty of Science, Institut Teknologi Sepuluh Nopember, Surabaya 60111, Indonesia

## Abstract

*Chromolaena odorata* L. (Asteraceae) is one of the tropical plants which is widely used as traditional medicines for diabetes and soft tissue wounds treatment in some regions in East Indonesia. The present study was aimed at determining the bioactive compounds of* C. odorata *leaves. The methanol and ethyl acetate extracts of* C. odorata* leaves have the inhibitory activity against 2,2-diphenyl-1-picryl-hydrazyl (DPPH) and 2,2'-azinobis-(3-ethylbenzothiazoline-6-sulfonic acid) (ABTS) radicals as well as *α*-glucosidase rat intestine enzyme. A new flavanone was isolated from the methanol extract and elucidated as 5,3'-dihydroxy-7,6'-dimethoxyflavanone or, namely, odoratenin (1) together with two known compounds: isosakuranetin (2) and subscandenin** (3)**. The antioxidant activity of odoratenin (1) exhibited very potent ABTS radical inhibitory activity with IC_50_ value of 23.74 *μ*M which is lower than that of trolox (IC_50_ 31.32 *μ*M) as a positive control. The result showed that a new flavanone, odoratenin (1), should be potential as an antioxidant source.

## 1. Introduction

Antioxidant is a bioactive substance preventing the oxidation of the harmful chemicals. That oxidation is caused by free radicals that have unpaired electrons. So, those free radicals are very reactive to damage molecules in cell [1]. During the past decade, a lot of antioxidant products are consumed by people in the world as the synthetic drugs, supplements, or traditional medicines. The traditional medicines have been taken by people in the world derived from the natural sources like medicinal plants according to World Health Organization (WHO) data. 65% of population in India consume the medicinal plants as a primary health. The 40% of prescription drugs in China are also based on the component of medicinal plants. In addition, 70% of Canadians have also used the medicinal plants as both a health supplement and an alternative therapeutic product [2]. In Indonesia, the medicinal plants are recognized as jamu. Approximately 85% of jamu's ingredients are the extract of medicinal plants. Hence, a number of modern or synthetic medicines are made from the isolation of natural sources based on the traditional plant medicines [3]. One of the natural sources that has been used as a medicinal plant is* Chromolaena odorata* L.

*C. odorata* (Asteraceae) is one of the species of* Chromolaena* genus that has been identified by King and Robinson in 1970.* C. odorata* is recognized as siam weed. It is one of the invasive species with a rapid growth forming the thick bushes as high as about two meters. Besides, it spreads rapidly on the open areas such as grasslands, roadsides, forests, nature reserves, and wildlife sanctuaries [4]. Actually,* C. odorata* is used as a medicinal plant by people lived in the tropic and subtropic areas. For example, in Vietnam, this plant is used as a treatment of leech bites, soft tissue injuries, burns, and skin infections [5]. Furthermore, a leaf water extract is widely used as a diarrhea, malaria, and diabetes drug [6]. Additionally, this leaf is also used as the treatment of wounds because the leaf's contents are protein, carbohydrate, and fiber source [7].

The previous studies have reported that most of the* Chromolaena* genus contains the flavonoids group. Based on a review information by Oliveira* et al*. (2017), they reported that about 40 flavonoids have been identified from this genus. One of species from this genus,* C. hirsuta*, has been reported to contain quercetin and kaempferol derivatives which belong to flavonoids group [8]. Some researchers also reported that* C. odorata *contains the flavonoid compounds [9–15]. In addition, the qualitative phytochemical properties of* C. odorata* leaves extract also showed the presence of secondary metabolite compounds such as coumarins, flavonoids, tannins, and sterols [16]. Currently, this preceding research aims to isolate and identify other secondary metabolite compounds of* C. odorata* leaves. Furthermore, the antioxidant activity of the compounds will be assayed.

Recently, some researchers reported that* C. odorata* showed bioactivity as an antibacterial [17], antifungal [18, 19], anti-inflammatory [20, 21], anticancer [11, 13, 22], antiplasmodial [9], antidiabetic [23, 24], and antioxidant [6, 25–28]. Rao* et al*. (2010) reported* in vitro* antioxidant activity of chloroform extract of* C. odorata* leves. The antioxidant activity was presented by using 2,2'-azinobis-(3-ethylbenzothiazoline-6-sulfonic acid) (ABTS) assay. The result showed a good inhibition with value of IC_50_ (1.32 mg/mL) compared to standard ascorbic acid (1.00 mg/mL) [28]. Furthermore, the antioxidant activity was also reported by ABTS assay from ethanol extract of* C. odorata*. The result showed a good amount of activity inhibition about 29.92-63.34% [26]. In addition, the significant activity was also obtained with polysaccharide fraction of* C. odorata* (91.91 ± 0.9%) by the same assay method [6]. However, its IC_50_ value, both research of Parameswari & Suriyavathana (2013) and Boudjeko* et al*. (2015), is not reported yet. Based on these studies,* C. odorata* has been recognized potentially as an antioxidant source. In the present study, the further research aims to identify the compounds of methanol extract from* C. odorata* leaves as an antioxidant.

## 2. Materials and Methods

### 2.1. Chemicals

The chemicals used were 2,2-diphenyl-1-picryl-hydrazyl (DPPH) (TCI, 1898-66-4), 2,2'-azinobis-(3-ethylbenzothiazoline-6-sulfonic acid) (ABTS) (Wako), pottasium peroxydisulphate (K_2_S_2_O_8_), Folin-Ciocalteu's phenol reagent (FCR) (Merck), anhydrous sodium carbonate (Na_2_CO_3_), rat intestinal acetone powder (Sigma, 1639), glucose kit liquor (HUMAN), acarbose, gallic acid, 6-hydroxy-2,5,7,8-tetramethylchromen-2-carboxylate acid (trolox) (Wako), and dimethylsulfoxide (DMSO) (Merck). Solvents (*n*-hexane, dichroloromethane, ethyl acetate, methanol, and ethanol) were purchased from Anhui Fulltime specialized solvents & reagents Co., Ltd. (Anhui, China).

### 2.2. General Experimental Procedures

The purity of the compounds was determined by column chromatography (CC) using silica gel 60 G (Merck), silica gel 60 (0,063-0,200 mm), and Sephadex LH-20. For thin layer chromatography (TLC) analysis, silica gel 60F_245_ aluminium sheets (Merck) were used. Spots were visualized under UV light and sprayed with CeSO_4_ in H_2_SO_4_ solution followed by heating. Fisher-Johns was used as melting point apparatus. The IR data were obtained on a Shimadzu FT-IR-8400S spectrometer using the KBr method. The 1D- and 2D-NMR, including ^1^H and ^13^C-NMR, HMBC (*Heteronuclear Multiple Bond Correlation*), and HMQC (*Heteronuclear Multiple Quantum Coherence*) spectra, were measured on a DELTA2_NMR spectrometer (JEOL, 400 MHz) with tetramethylsilane as a standard in CDCl_3_. The molecular formula was confirmed by using Xevo G2-XS QTof LC-MS-MS with ESI for type of ionization. The absorbance data were measured on UV-Vis Genesys Thermo Scientific 10S spectrophotometer.

### 2.3. Plant Material

The leaves of* C. odorata *were collected on August 2017 at Ambon, Maluku Province, Indonesia. The plant was identified with a voucher specimen (48) by Pamela Papila, a botanist at the Fundamental Biology Laboratory, Pattimura University, Indonesia.

### 2.4. Extraction

The dried leaves of* C. odorata* (30 g) were extracted with various solvents for the bioactivity preparation assay. The leaves were dried in room temperature. They were extracted by using* n*-hexane, dichloromethane, ethyl acetate, methanol, and water in 200 mL of solvent for each extract at room temperature for 24 hours. The solvent was removed from the extracts by rotary evaporator to obtain the five crude extracts.

### 2.5. Fractionation

The dried leaves of* C. odorata* (2.76 kg) were extracted during 3 × 24 hours at room temperature in 10 L MeOH for each time. The solvent was removed from the extract by rotary evaporator to yield 832 g of extract (30.15% yield). 90 g of methanol extract was then fractionated by CC vacuum on silica gel 60 G (480 g) with a gradient elution of CH_2_Cl_2_ (100%), EtOAc (100%), and MeOH (100%), each 5.4 L to obtain three fractions (A-C). Fraction A (25.6 g) was further subjected to CC vacuum (Si gel 60 G, 180 g) with a step gradient elution of* n*-hexane:EtOAc (99:1, 97:3, 93:7, 90:10, 75:25, 50:50, 25:75, and 0:100,* v/v*, each 900 mL) and MeOH to obtain five subfractions (A1-A5). Subfraction A3 (6.4 g) was subjected to CC vacuum (Si gel 60 G, 92 g) with a step gradient elution of* n*-hexane:EtOAc (93:7, 92:8, 91:9, 90:10, 88:12, 86:14, 82:18; 80:20, 50:50, 20:80, and 0:100,* v/v*, each 500 mL) and MeOH, respectively, to obtain eight subfractions (A3A-A3H). Furthermore, subfraction A3E (1.7 g) was subjected to Sephadex LH-20 CC eluted with CH_2_Cl_2_:MeOH (1:1,* v/v*) to yield five subfractions (A3E1-A3E5). Subfraction A3E4 (0.6 g) was subjected to Sephadex LH-20 CC eluted with CH_2_Cl_2_:MeOH (1:1,* v/v*) to obtain four subfractions (A3E4A-A3E4D). A compound (2) (29.8 mg) was obtained by purification of subfraction A3E4C (100 mg) with recrystallization technique. Furthermore, subfraction A3E4B (400 mg) was separated by using silica gel 60 CC (50 g) eluted with CH_2_Cl_2_ (100%) to afford compound (1) (54.1 mg) and six subfractions (A3E4B1-A3E4B6). The compound of (1) was obtained by purification of subfraction A3E4B1 (100 mg). And compound** (3)** (5 mg) was obtained by purification of subfraction A3E4B2B2B3 (80 mg).

### 2.6. Antioxidant Activity

#### 2.6.1. Determination of Total Phenolic Contents

The total phenolic content of various* C. odorata* extracts (the* n*-hexane, dichloromethane, ethyl acetate, methanol, and water extracts) was determined according to the procedure of Qassabi* et al*. (2018) with slight modifications [29]. The total phenolic content was determined by applying gallic acid calibration curve and expressed in mg of gallic acid equivalents (GAE)/g crude extracts. Each extract (1 mg/mL) was dissolved in methanol to prepare a sample solution. The absorbance of sample solution was determined by using UV-Vis Genesys Thermo Scientific 10S spectrophotometer with those following steps. First, the mixture solution between 66 *μ*L of sample solution and 500 *μ*L of 10% FCR solution was mixed and incubated in a dark place for 5 minutes. Then, 500 *μ*L of 6% Na_2_CO_3_ was added into the solution, mixed well, and left for 90 minutes in the dark place. Finally, the absorbance of sample solution was measured by UV-Vis spectrophotometer at *λ* 750 nm.

#### 2.6.2. DPPH Radical Scavenging Assay

DPPH assay was performed based on the method published previously [30]. First, DPPH solution (6 × 10^−5^ M) was separated by dissolving 2.37 mg of DPPH in 100 mL of methanol to obtain a working solution. Then, 1 mL the working solution was mixed with 33 *μ*L of samples (*n*-hexane, dichloromethane, ethyl acetate, methanol, and water extracts) at maximum dissolved concentration in methanol and mixed well. Finally, the mixed sample solution was incubated for 20 minutes at room temperature. Then, the absorbance (A_s_) of the reaction mixture was measured by UV-Vis spectrophotometer at 517 nm. The mixed solution between methanol and the working solution was used as blank to give the blank absorbance (A_b_). Gallic acid was used as a standard. The inhibitory activity was calculated by (1). The IC_50_ value is expressed as a quantity of an extract inhibitory concentration against a half of DPPH radicals.

(1) $$\begin{array}{c} Inhibition\left(\;\%\;\right)=\left[\;\frac{\left(A_{b}-A_{s}\right)}{A_{b}}\;\right]\times 100 \end{array}$$

#### 2.6.3. ABTS Radical Cation Scavenging Assay

Free radical scavenging by ABTS radical was based on the method described previously by us [30]. First, ABTS solution (7 mM) was prepared by dissolving 19.2 mg of ABTS in 5 mL of water and, then, 140 mM K_2_S_2_O_8_ in 88 *μ*L of water. Those two solutions were mixed and incubated for 12-16 hours to obtain ABTS radical cation solution which is a dark blue solution. It was added with ± 274 mL of ethanol to give an absorbance of 0.7 ± 0.02 units at 734 nm for making a working solution. 1 mL of working solution was mixed with 10 *μ*L of samples (*n*-hexane, dichloromethane, ethyl acetate, methanol, and water extracts) at maximum dissolved concentration in DMSO and mixed well. Finally, the mixed sample solution was incubated for four minutes at room temperature; then, the absorbance (A_s_) of the reaction mixture was measured by UV-Vis spectrophotometers at 734 nm. The mixed solution between DMSO and the working solution was used as blank to give the blank absorbance (A_b_). Trolox was used as a standard. The inhibitory activity was calculated by (1). The IC_50_ value was expressed as a quantity of an extract inhibitory concentration against a half of ABTS radicals.

### 2.7. *α*-Glucosidase Inhibitory Activity Assay

The *α*-glucosidase inhibitory assay was performed based on the procedure from Ayinampudi* et al*., (2012) with some modifications [31]. First, rat intestinal acetone powder (1 g) was suspended in 30 mL of normal saline. This suspended solution was sonicated for five minutes at 4°C. After centrifugation (12,000 rpm, 30 minutes, 4°C), the resulting supernatant was used for the assay. Briefly, a mixture of 10 *μ*L samples, 30 *μ*L of 0.1 M phosphate buffer (pH 6.9), 20 *μ*L of 10 mM maltose, 80 *μ*L glucose kit, and 20 *μ*L of enzyme supernatant were incubated in 96-well plates at 37°C for 10 minutes. Acarbose was used as a standard. The absorbance was recorded at 490 nm by microplate reader (Biotek ELx800UV). The inhibitory activity was determined from the formula as follows:

(2) $$\begin{array}{c} Inhibition\left(\;\%\;\right)=\left[\;\frac{\left(A_{blank}-A_{sample}\right)}{A_{blank}}\;\right]\times 100 \end{array}$$

where A_blank_ = A_enzyme reaction_ − A_blank of enzyme reaction_ and A_sample_ = A_sample reaction_ − A_blank of sample reaction_.

## 3. Results

### 3.1. Extraction

The five crude extracts from* C. odorata* leaves have been obtained. The methanol extract has the highest yield of all extracts. From 30 g dried leaves in 200 mL of each solvent, the yields of the five extracts were obtained such as 4.33% yield of* n*-hexane, 6.77% yield of dichloromethane, 7.33% yield of ethyl acetate, 10.00% yield of methanol, and 7.33% yield of water extract.

### 3.2. Total Phenolic Content

The total phenolic content of different extracts of* C. odorata* leaves was determined by using FCR according to the procedure of Qassabi* et al*. (2018) with slight modifications. The tested extracts are* n*-hexane, dichloromethane, ethyl acetate, methanol, and water extracts at concentration 61.91 *μ*g/mL. The evaluated result of total phenolic content of each extract is showed in Table 1. Gallic acid was used as a standard for calibration curve to determine the amount of total phenolic content. Based on study, the total phenolic content of different extracts varied from 14.65 to 104.08 *μ*gGAE/mg of extract. The ethyl acetate extract is the highest amount of total phenolic content of all the extracts with value of 104.08 *μ*gGAE/mg of ethyl acetate extract.

### 3.3. DPPH Radical Scavenging Activity

**Figure 1:**
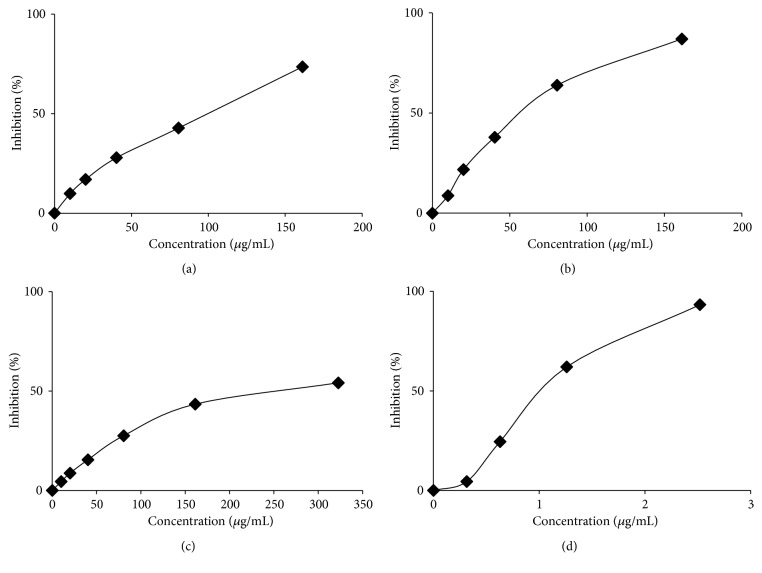
DPPH radical scavenging activity of* C. odorata *(a) dichloromethane, (b) ethyl acetate, (c) methanol extracts, and (d) gallic acid as a standard.

DPPH radical scavenging activity of the five extracts and gallic acid as a standard are presented in Figure 1 and summarized in Table 1. Based on these IC_50_ values, the dichloromethane, ethyl acetate, and methanol extracts are potential antioxidant against DPPH radicals with IC_50_ value of 90.83, 57.26, and 188.61 *μ*g/mL, respectively. According to this result, the ethyl acetate extract is the highest inhibitory activity against DPPH radicals among other extracts. The minimum of IC_50_ value indicates a good free radical scavenging activity.

### 3.4. ABTS Radical Cation Scavenging Activity

**Figure 2:**
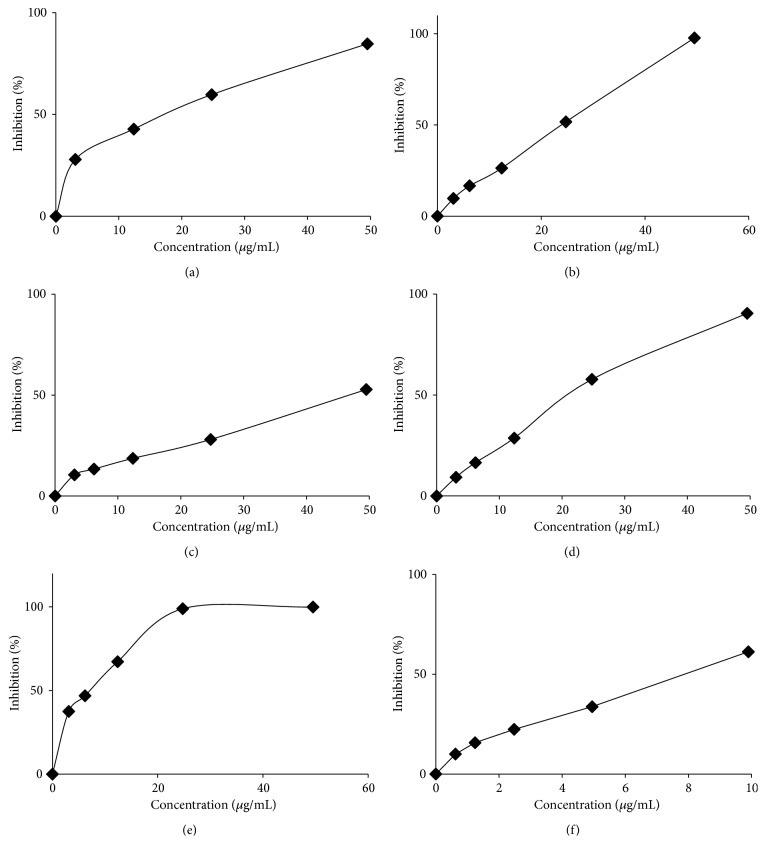
ABTS scavenging activity of* C. odorata *(a) dichloromethane, (b) ethyl acetate, (c) methanol, (d) water extracts, (e) trolox as a standard, and (f) odoratenin (1).

ABTS radical cation scavenging activity of the five extracts, compounds isolated from* C. odorata*, and trolox as a standard are presented in Figure 2 and summarized in Table 1. According to these IC_50_ values, both the extracts and compounds are potential antioxidant against ABTS radical cations. The IC_50_ values of dichloromethane, ethyl acetate, methanol, and water extracts are 13.97, 24.43, 46.79, and 21.37 *μ*g/mL, respectively. Interestingly, odoratenin (1) is higher free radical scavenging activity than that of the various extracts with IC_50_ value of 7.51 *μ*g/mL (23.74 *μ*M) also compared with trolox as a standard with IC_50_ value of 31.32 *μ*M.

### 3.5. *α*-Glucosidase Inhibitory Activity

**Figure 3:**
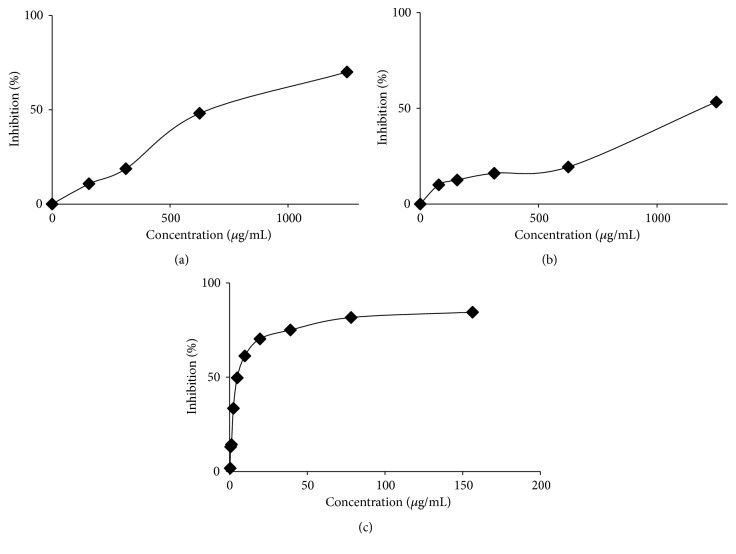
*α*-Glucosidase inhibitory activity of* C. odorata *(a) ethyl acetate, (b) methanol extracts, and (c) acarbose as a standard.

Rat intestinal acetone powder was used in this assay system. The five extracts, compounds isolated from* C. odorata*, and acarbose as a standard are showed in Figure 3 and summarized in Table 1. In this assay system, the ethyl acetate extract was found to be slightly more active than that of the methanol extract. In contrast, the* n*-hexane, dichloromethane, water, and the compounds** (1-2)** had a weak effect on the enzyme activity. The ethyl acetate extract presented the inhibitory activity with an IC_50_ value of 779.54 *μ*g/mL. Acarbose, which is known as a potent *α*-glucosidase inhibitor, was used as a standard and showed an IC_50_ of 7.67 *μ*g/mL in our assay system.

### 3.6. Odoratenin (**1**)

**Figure 4:**
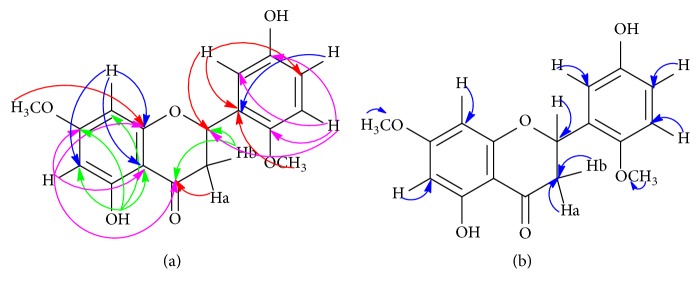
(a) HMBC and (b) HMQC correlations of odoratenin (1).

White crystal; mp: 144-145°C; [*α*]_25_^*D*^ -16.0° (CHCl_3_;* c* = 0.001); IR *ν*_max_ (KBr): 3518, 2943, 1629, 1593, 1519, 1442, 1300, 1278, 1201, 810 cm^−1^; for ^1^H (400 MHz, CDCl_3_), ^13^C NMR (400 MHz, CDCl_3_), HMQC, and HMBC spectroscopic data are presented in Figure 4 and summarized in Table 2; and HR-ESI-MS* m/z* 339.0831 [M + Na]^+^ (cald. for C_17_H_16_O_6_Na, 339.3090).

### 3.7. Isosakuranetin (**2**)

White needles; mp: 173-174°C; IR *ν*_max_ (KBr): 3504, 2955, 1639, 1599, 1518, 1492, 1302, 1253, 1163, 833 cm^−1^; for ^1^H (400 MHz, CDCl_3_) and ^13^C NMR (400 MHz, CDCl_3_) spectroscopic data are presented in Table 2.

### 3.8. Subscandenin (**3**)

Yellow needles; mp: 174-175°C; for ^1^H (400 MHz, CDCl_3_), ^13^C NMR (400 MHz, CDCl_3_) and HMBC spectroscopic data are presented in Table 2.

## 4. Discussion

### 4.1. Antioxidant Activities of* C. odorata*

*C. odorata* is a species of the genus* Chromolaena* which is one of the largest genera of the family Eupatorieae (Asteraceae) [8]. In Indonesia,* C. odorata, *known as sungga-sungga, was collected from Ambon, Maluku, East Indonesia. This plant is a popular folk medicine widely used as alternative herbal treatment for diabetes and soft tissue wounds in East Indonesia. Besides, in Vietnam, this plant is used as a treatment of leech bites, soft tissue injuries, burns, and skin infections [5]. Furthermore, a leaf water extract is widely used as a diarrhea, malaria, and diabetes drug [6]. In the past 40 years, this plant has been reported in phytochemical studies in the United States [14, 15]. Recently,* C. odorata* was described for its beneficial attributes in some Asia-Africa countries, especially the pharmacological effects of this plant. The specific reported attributes of* C. odorata* include being antibacterial [17], antifungal [18, 19], anti-inflammatory [20, 21], anticancer [11, 13, 22], antiplasmodial [9], antidiabetic [23, 24], and antioxidant [6, 25–28]. However, the antioxidant activity of the isolated compound from* C. odorata* has never been reported.

This present study demonstrated the antioxidant activity of the isolated compound from* C. odorata* for the first time. Related to this study, the antioxidant effect from this plant has been done by two radical scavenging assays supported with the total phenolic content data. As we know, there are a lot of free radical types caused of reactive oxygen species (ROS) [32]. They are the dangerous free radicals against the human body. These free radicals come from either the body itself or the external factors. The free radicals are by products of energy production by mitochondria which are energy-producing cells as adenosine triphosphate (ATP), while the external factors come from the pollutions, ultraviolet radiation, diet, or lifestyle. Furthermore, the free radicals have an unpaired electron so this condition makes them to be reactive with other molecules around them [33]. Then, the molecules in cells are attacked by free radicals. Normally, the body's antioxidant defence system can block the free radicals before they become harmful to the body. Unfortunately, because of the old age or a lot of toxin that accumulate inside, the defence system works slowly and then the free radicals start to cause cell damage. The cell damage caused by free radicals is called oxidative stress. On the long term, the danger of free radicals inside is related to aging and chronic diseases such as cancer, diabetes, and neurodegenerative and cardiovascular diseases.

In the body, free radicals are superoxide anion (SOA) from 2.5% oxygen (O_2_). The using of O_2_ in the body as a distributor of energy products changes because of free radicals of SOA. Because of that, the body is protected from those free radicals by its antioxidant defence system with these following steps [34]. First, SOA is neutralized by the antioxidant enzyme superoxide dismutase (SOD) changed as hydrogen peroxide (H_2_O_2_). H_2_O_2_ is a weak free radical which is used as an immune compound to inhibit the pathogen bacteria or to treat the broken cell tissue. However, the large amounts of H_2_O_2_ will be toxic for the body. So, there is the second step from the body's defence system that helped with glutathione peroxidase (GPO) enzyme. Two GPOs covert H_2_O_2_ into two water molecules (H_2_O). Certainly, H_2_O is safer than that of H_2_O_2_. Those two steps are very important to protect the cell body. Unfortunately, there is not enough amount of SOD and GPO in the body. So, the amount of free radicals of SOA and H_2_O_2_ will increase in the cell. The SOA and H_2_O_2_ might not be worse. But in excess, they will react with each other into more dangerous free radicals, namely, hydroxyl radicals (•OH). Hence, an antioxidant is needed as a resistance support from the outside of the body's defence system [35].

Studies are in our laboratory to identify the antioxidant compound present in* C. odorata*. The determination of the antioxidant effect was assayed by using DPPH and ABTS radicals. As we described previously, there are a lot of free radical types caused by ROS including DPPH and ABTS radicals. DPPH radicals are expressed as the free radical with high reactivity at room temperature. The high reactivity is caused by delocalization of electrons around the molecules. The mechanism of radical scavenging is hydrogen donors. When the DPPH radical is reacted with a substance that donates a hydrogen atom, DPPH radical is reduced into a nonradical DPPH. In the assay, this reaction is characterized by decolorization of the solution. It changes its colour solution from purple to yellow. At room temperature, ABTS radical is more stable and has higher reactivity than that of DPPH. The ABTS radicals are expressed as cation radical with high reactivity ability [36]. The radical cation is formed from the oxidation reaction between ABTS and buffer solution especially using K_2_S_2_O_8_. Furthermore, the mechanism of radical scavenging as well as DPPH's mechanism is hydrogen donors [35]. Thus, the antioxidant activity for both of the two assays is evaluated by using UV-Vis Genesys Thermo Scientific spectrophotometer.

According to this study, there is a linear relationship between the antioxidant activity and total phenolic content. This evidence means that the higher the total phenolic content, the higher the antioxidant activity. Among the five tested extracts, the ethyl acetate extract exhibited the highest antioxidant activity against either DPPH or ABTS because of the high amount of total phenolic content. Not only the ethyl acetate extract, but also the dichloromethane, methanol, and water extracts, showed fine antioxidant activity as well as the ethanol and chloroform extracts reported previously [27, 28]. However, there was only weak activity in the* n*-hexane extract. These results suggest the presence of phenolic compounds could be major contributor to antioxidant activity. The phenolic compounds including xanthone [37] or stilbene [38] have been reported as a potent antioxidant activity. Based on this study, the phenolic compounds of* C. odorata* could be extracted by the polar and semipolar solvents very well. When the methanol extract was fractionated and elucidated, the major secondary metabolite came from flavanone compounds which is one of the phenolic groups. The finding of a new flavanone, odoratenin (1), indicates the presence of two hydroxyls and two methoxyl group. They might be donated and the hydrogen atom also supported the electron conjugation system from the phenolic ring for stabilizing the free radicals. Interestingly, the new compound odoratenin (1) has higher antioxidant activity than that of trolox as a standard. The present study and these results reveal odoratenin (1) isolated from* C. odorata* as a potent antioxidant source.

### 4.2. *α*-Glucosidase Inhibitory Activity of* C. odorata*

Diabetes mellitus is a metabolic disorder caused by a lack of insulin [39]. Insulin helps the blood glucose level to be a normal circumstance and not turn into hyperglycemia or hypoglycaemia. According to the type of an abnormal insulin, diabetes mellitus is divided into two types [40]. The first type is known as insulin dependent diabetes mellitus (IDDM) caused by a genetic factor such as the destruction of pancreatic *β*-cells which produce insulin and type 2 is non-insulin dependent diabetes (NIDDM) caused by a wrong lifestyle especially on diet. This study focuses on the effective treatment for type 2. As we know, carbohydrates are the major components of our daily foods, for instance, polysaccharides or disaccharides. After carbohydrates intake, the amount of polysaccharides is transformed into monosaccharides as known as the simple sugars, and then they are transferred through the bloodstream system for energy [41]. However, before they are transferred, they are absorbed on the intestine. In the small intestinal tissue, there is a catalyse of the cleavage of polysaccharides to glucose, namely, *α*-glucosidase. It makes the total of glucose too large. Certainly, the increasing of glucose level as known as hyperglycemia in the blood is not good enough for health [42, 43]. Related to hyperglycemia, *α*-glucosidase inhibitor is recommended as antidiabetic [44].

In this assay system, the rat intestinal acetone powder has been used as enzyme to determine the antidiabetic inhibitory activity of* C. odorata* extracts. The tested extracts might inhibit or compete with maltose as a substrate. Based on our work, there is a linear correlation between *α*-glucosidase inhibition and antioxidant activity. The ethyl acetate and methanol extracts performed a fine inhibitory activity. This result as well as the antioxidant activity screening previously reported that these two extracts have a good radical scavenging activity also against both DPPH and ABTS radicals. Furthermore, the isolated compound from* C. odorata* reported a significantly higher *α*-glucosidase inhibitory activity than that of deoxynojirimycin and acarbose as the standards in previous research [24]. From this study, it should be noted that the *α*-glucosidase inhibitory effect of the ethyl acetate extract from* C. odorata* was almost the same as that of the methanol extract.

### 4.3. The Flavanones Isolated from* C. odorata*

Antioxidant evaluation of the methanol extract from the leaves of* C. odorata* led to finding of a bioactive compound as well as a new flavanone, odoratenin (1), along with two known compounds: isosakuranetin (2) and subscandenin** (3)**. The structure of a new compound (1) was elucidated by using 1D- and 2D-NMR spectroscopy analysis and the structures of the known compounds (2) and** (3)** were determined and compared with the published NMR spectroscopic data previously.

**Figure 5:**
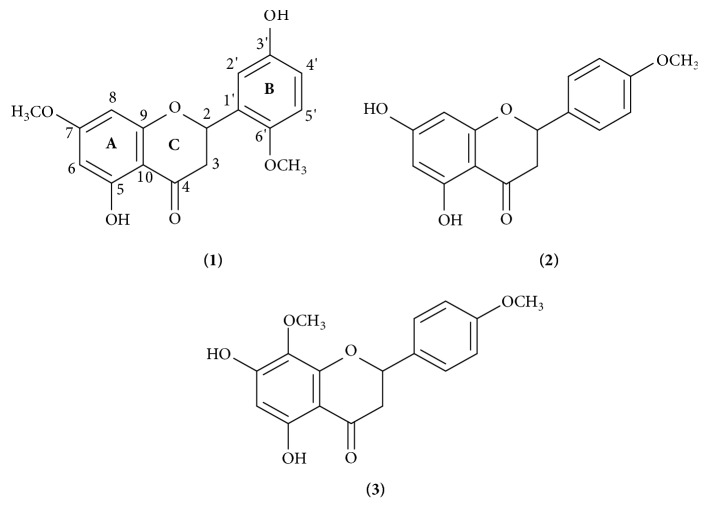
The structures of odoratenin (1), isosakuranetin (2), and subscandenin** (3)** isolated from the leaves of* C. odorata* methanol extract.

Odoratenin (1) was obtained as a white crystal. Its molecular formula was determined as 5,3'-dihydroxy-7,6'-dimethoxyflavanone (C_17_H_16_O_6_) by HR-ESI-MS measurement through the hydrogen ion at* m/z* 317.1013 [M + H]^+^ and the sodium ion at* m/z* 339.0831 [M + Na]^+^ (calcd. for C_17_H_16_O_6_Na, 339.3090). The IR spectrum showed characteristic absorption bands for hydroxyl chelated carbonyl stretching bonds at 3518 and 1629 cm^−1^, indicating the presence of a flavonoid group. The NMR assignments were made by applying 1D- and 2D-NMR experiments (^1^H NMR, ^13^C NMR, HMBC, and HMQC; CDCl_3_, 400 MHz). The ^1^H NMR spectrum (Table 2) showed signal for a hydrogen-bonded hydroxyl proton at *δ*_H_ 12.01 (s, 1H, 5-OH); two aromatic protons at *δ*_H_ 6.06 (d,* J*=2.8 Hz, 1H, H-6) and 6.04 (d,* J*=2.4 Hz, 1H, H-8); three pyrone vicinal-geminal protons at *δ*_H_ 5,32 (dd,* J*=12.8; 3.2 Hz, 1H, H-2), 3.07 (dd,* J*=17.2; 12.8 Hz, 1H, H-3b), and 2.77 (dd,* J*=17.0; 3.2 Hz, 1H, H-3a) indicating the presence of a flavanone which are similar to the ^1^H NMR spectrum of known compound, isosakuranetin (2); three aromatic protons with abx-system at *δ*_H_ 7.04 (d,* J*=2.0 Hz, 1H, H-2'), 6.91 (dd,* J*=1.6 Hz, 1H, H-4'), and 6.88 (d,* J*=8.4 Hz, 1H, H-5'); and six methoxy protons at *δ*_H_ 3.92 (s, 3H, 6'-OMe) and 3.82 (s, 3H, 7-OMe). Based on the ^13^C NMR spectrum, there are 17 carbon signals of this compound (1) including a carbonyl group of C-4 (*δ*_C_ 196.07), one chiral carbon of C-2 (*δ*_C_ 79.06) with [*α*]_25_^*D*^ -16.0° as the absolute configuration, two carboxyl groups (-C-OH) of C-5 and C-3' (*δ*_C_ 162.93 and 145.99), and two methoxy carbons (-OCH_3_) of 7-OCH_3_ and 6'-OCH_3_ (*δ*_C_ 55.79 and 56.14). However, there is the only one methoxy carbon of 4'-OCH_3_ (*δ*_C_ 55.48) in isosakuranetin (2). The long-range hydrogen to carbon correlations were assigned and confirmed by two-dimensional NMR (HMBC and HMQC) as Figures 4(a) and 4(b). The HMBC correlations showed that a hydrogen-bonded hydroxyl proton at *δ*_H_ 12.01 (5-OH) correlated with C-6 (*δ*_C_ 95.19), C-7 (*δ*_C_ 164.19), C-8 (*δ*_C_ 94.33), and C-10 (*δ*_C_ 103.20) showing that the hydroxyl group was attached to C-5. Two vicinal-geminal protons at *δ*_H_ 3.07 (3-Hb) and 2.77 (3-Ha) were attached to carbon carbonyl (C-4, *δ*_C_ 196.07). Besides, the 3-Hb proton correlated with C-2 (*δ*_C_ 79.06) showing that the vicinal-geminal protons were attached to C-3. Furthermore, five aromatic protons were placed at C-6, C-8, C-2', C-4', and C-5'. They have hydrogen-to-carbon correlations between H-6/C-4, C-7, C-9, C-10; H-8/C-6, C-7, C-9, C-10; H-2'/C-2, C-1', C-4'; H-4'/C-1'; H-5'/C-2, C-2', C-3', C-6' which were also confirmed by HMQC spectrum as Figure 4(b). Accordingly, both hydroxyl and methoxy substituents were assigned as 3'-hydroxy [9, 13] and 8-methoxy [14] at C-3' and C-8. Interestingly, although more than 79 flavonoid compounds have been isolated from the genus* Chromolaena *[8], the methoxy substituent at C-6' has not been reported yet before. Accordingly, the compound of (1) is a new flavanone named odoratenin (1) as in Figure 5(1).

Isosakuranetin (2) is a white needles solid powder with a melting point of 173-174°C. The elucidation process of isosakuranetin (2) was determined as these following steps. First, the FT-IR data showed the strong intensity of peaks as follows, *ν*_maks_ (KBr): 3504, 2955, 1639, 1599, 1518, 1492, 1302, 1253, 1163, and 833 cm^−1^. The peaks of 3504 cm^−1^ with medium intensity and 1639 cm^−1^ with strong intensity revealed the presence of a hydroxyl (-OH) group chelated with carbonyl group (-C=O). In addition, the peaks of 1518, 1492, and 833 cm^−1^ with medium to weak intensities indicated the presence of a conjugated aromatic group. Furthermore, this information was confirmed by data ^1^H and ^13^C-NMR (CDCl_3_, 400 MHz) presented in Table 2. Based on the presented data, there are a number of detected chemical shifts as 14 protons and 16 carbons. The 14 proton signals including the singlet signal of *δ*_H_ 12.04 ppm with integration in the downfield area indicated the presence of one proton as 5-OH. This proton is deshielded because it bonds with O atom which has more electrons directly to be a hydroxyl group and also as a typical signal at the same time. The typical signal means a hydroxyl proton chelated with a carbonyl group. So, the proton has a far chemical shift. Next, a strong singlet signal of *δ*_H_ 3.83 ppm with three integration processes in the upfield area showed three protons as 4'-OCH_3_. They are shielded because they do not bond with O atom directly. So, these three protons have near chemical shifts and also they are suspected strongly to be protons from the methoxy group. Furthermore, three signals with doublet multiplicity at *δ*_H_ 5.36 (H-2), 2.78 (H-3a), and 3.09 ppm (H-3b) coupling with the vicinal-geminal proton system indicated the presence of a pyran group [45]. This proton system indicated strongly that compound (2) is one of the flavanone groups which is similar to a previous known compound reported by Suksamrarn* et al*. (2004) [13]. In addition, the doublet signals of *δ*_H_ 7.37 (H-2'/6') and 6.95 ppm (H-3'/5') coupling with each other with double intensity showed four proton signals indicating the protons of an aromatic group. Based on our results, compound (2) has a flavanone skeleton with an ABC ring system substituted with the methoxy and hydroxyl groups as Figure 5(2). Furthermore, two signal doublets of *δ*_H_ 5.97 and 5.99 ppm also coupling with each other are strongly suspected as two signals of aromatic potons as H-6 and H-8 on ring A. The determination of the structure of compound (2) is confirmed with ^13^C-NMR data. Based on our ^13^C-NMR data, there are 16 carbons including a carbon carbonyl group which is strongly expected as a position of C-4 (*δ*_C_ 196.18 ppm), one chiral carbon assumed as a position of C-2 (*δ*_C_ 79.10 ppm), one carbon methoxy at position of C-4' (*δ*_C_ 55.48 ppm), and the aromatic carbon expected as the position of C-2'/6' (*δ*_C_ 114.33 ppm) and C-3'/5' (*δ*_C_ 127.85 ppm) with double intensity, respectively. And the other aromatic carbons including *δ*_C_ 95.54, 96.76, 103.20, 130.36, 160.14, 163.36, 164.53, and 164.59 ppm. Based on this elucidation study, compound (2) is an isosakuranetin (2) as in Figure 5(2) which was also isolated by Suksamrarn* et al*. (2004).

Subscandenin** (3)** is yellow needles solid powder with a melting point of 174-175°C. Compound** (3) **is strongly expected as subscandenin which is one of the derivatives from flavanone compounds with a skeleton similar to compounds** (1-2)**. The structure is confirmed by the interpretation of ^1^H, ^13^C-NMR, and HMBC data (CDCl_3_, 400 MHz) presented in Table 2. The results of the NMR characterization showed characters that are similar to the NMR characterization of compounds** (1-2)**. Based on the presented data, there are a number of detected chemical shifts as 16 protons and 17 carbons. This total of protons and carbons is equal to the total of compound (1). The ^1^H NMR spectrum (Table 2) showed signal for a hydrogen-bonded hydroxyl proton at *δ*_H_ 12.19 (s, 1H, 5-OH); two aromatic protons at *δ*_H_ 6.10 (s, 1H, H-6) and 6.46 (br s, 1H, H-7); three pyrone vicinal-geminal protons at *δ*_H_ 5.34 (dd,* J*=13.2; 3.2 Hz, 1H, H-2), 3.07 (dd,* J*=17.2; 12.8 Hz, 1H, H-3b), and 2.77 (dd,* J*=17.2; 3.2 Hz, 1H, H-3a) indicating the presence of a flavanone which are similar to the ^1^H NMR spectrum of the known compounds, odoratenin (1) and isosakuranetin (2); four aromatic protons at *δ*_H_ 6.95 (d,* J*=10 Hz, 2H, H-2'/6') and 7.37 (d,* J*=9.2 Hz, 2H, H-3'/5'); and six methoxy protons at *δ*_H_ 3.94 (s, 3H, 8-OMe) and 3.82 (s, 3H, 4'-OMe). Based on the ^13^C NMR spectrum, there are 17 carbon signals of compound** (3)** including a carbonyl group (*δ*_C_ 196.96), one chiral carbon (*δ*_C_ 79.13), two carboxyl groups (*δ*_C_ 158.78 and 114.30), and two methoxy carbons (*δ*_C_ 61.09 and 55.46). The long-range hydrogen to carbon correlations were assigned and confirmed by HMBC spectrum presented in Table 2. The HMBC correlation data confirmed the existence of 18 correlations between protons and carbons. These results showed the structure of compound** (3)** is different from either compound (1) or compound (2). The signal correlation showed a relationship between proton methoxy of 4'-OCH_3_ (*δ*_H_ 3.82 ppm) with carbon of C-4 (*δ*_c_ 160.13 ppm) and other proton methoxy of 8-OCH_3_ (*δ*_H_ 3.94 ppm) with two carbons of C-4 (*δ*_c_ 196.96 ppm) and C-8 (*δ*_c_ 128.38 ppm). This signal correlation revealed the position of the methoxy of 4'-OCH_3_ (*δ*_H_ 3.82 ppm) on ring B and other methoxy group of 8-OCH_3_ (*δ*_H_ 3.94 ppm) on ring A. Furthermore, there are four correlations between protons and aromatic carbon on ring B, namely, proton of H-3'/5' (*δ*_H_ 7.37 ppm) correlating with four carbons of C-1 (*δ*_c_ 130.36 ppm), C-2'/6' (*δ*_c_ 127.81 ppm with double integrations), and C-4' (*δ*_c_ 160.13 ppm). The same thing happened to proton of H-2'/6/ (*δ*_H_ 6.95 ppm) which also correlated with four carbons of C-1' (*δ*_c_ 130.36 ppm), C-3'/5' (*δ*_c_ 114.30 with double integrations), and C-4' (*δ*_c_ 160.13 ppm). These correlation evidences assumed strongly that the O-methoxy binds directly to carbon of C-4'. In addition, there are four correlations between a proton and the aromatic carbons on ring A, namely, proton of H-6 (*δ*_H_ 6.10 ppm) correlating with four carbons of H-5 (*δ*_c_ 158.78 ppm), H-8 (*δ*_c_ 128.38 ppm), H-9 (*δ*_c_ 157.50 ppm), and H-10 (*δ*_c_ 103.19 ppm). These correlation evidences assumed strongly that the O-methoxy binds directly to carbon of C-8. Based on our elucidation study, the compound of** (3)** is a subscandenin** (3)** as in Figure 5**(3)** which was also isolated by Amaro-Luis & Delgado-Mendez (1993). However, they reported isolated subscandenin** (3) **from a different species, namely,* C. subscandens* [14].

## 5. Conclusion

*C. odorata*, collected from East Indonesia, contributes to drug discovery and healthcare. This is the first report on the antioxidant activity of a new flavanone isolated from the* C. odorata* leaves methanol extract. Among the tested five extracts, the ethyl acetate extract exhibited the highest inhibitory effect against ABTS radical and *α*-glucosidase rat intestinal enzyme. Further investigations will focus on the identification of the other active flavanone compounds responsible for the antioxidant as well as *α*-glucosidase inhibitory activity of* C. odorata* leaves ethyl acetate extract.

## Figures and Tables

**Table 1:** Antioxidant and *α*-glucosidase activities of the extracts and compounds from *C. odorata*.

Samples	Total phenolic contents (*μ*g GAE/mg of sample) ± SD^1^	DPPH IC_50_ (*μ*g/mL) ± SD^1^	ABTS IC_50_ (*μ*g/mL) ± SD^1^	*α*-Glucosidase inhibition IC_50_ (*μ*g/mL) ± SD^1^
Samples (Extracts of *C. odorata*)				

*n*-Hexane	14.65 ± 0.98	>319.46	>99.01	>1250
Dichloromethane	74.84 ± 2.11	90.83 ± 0.31	13.97 ± 0.22	>1250
Ethyl acetate	104.08 ± 3.87	57.26 ± 1.07	24.43 ± 0.09	779.54 ± 6.16
Methanol	57.11 ± 4.85	188.61 ± 3.31	46.80 ± 2.91	1329.31 ± 2.68
Water	27.49 ± 1.41	>319.46	21.37 ± 0.89	>1250

Samples (the compounds isolated from *C. odorata*)				

Odoratenin (1)	NS^2^	NS^2^	7.51 ± 1.57	>62.5
Isosakuranetin (2)	NS^2^	NS^2^	>9.9	>312.5
Subscandenin (3)	NS^2^	NS^2^	NS^2^	NS^2^

Standard				

Gallic acid	as a standard curve	1.11 ± 0.42	NS^2^	NS^2^
Trolox	NS^2^	NS^2^	7.84 ± 0.45	NS^2^
Acarbose	NS^2^	NS^2^	NS^2^	7.67 ± 1.86

^1^values represent the means ± standard deviations for triplicate experiments.

^2^not studied.

**Table 2:** 1D- and 2D NMR spectroscopic data of compounds **(1-3) **in CDCl_3_.

Position	Isosakuranetin (2)		Odoratenin (1)			Subscandenin (3)		
	*δ*_H_ (*J* in Hz)	*δ*_c_	*δ*_H_ (*J *in Hz)	*δ*_c_	HMBC	*δ*_H_ (*J *in Hz)	*δ*_c_	HMBC
2	5.36 (dd; *J*=13.2, 3.2 Hz, 1H)	79.10	5.32 (dd; *J*=12.8, 3.2 Hz, 1H)	79.06	-	5.34 (dd; *J*=13.2, 3.2 Hz, 1H)	79.13	-
3a	2.78 (dd; *J*=17.8, 3.2 Hz, 1H)	43.20	2.78 (dd; *J*=17.0, 3.2 Hz, 1H)	43.30	C-4	2.77 (dd; *J*=17.2, 3.2 Hz, 1H)	43.29	C-4
3b	3.09 (dd; *J*=17.0, 13.2 Hz, 1H)		3.07 (dd; *J*=17.2, 12.8 Hz, 1H)		C-2, 4	3.07 (dd; *J*=17.2, 12.8 Hz, 1H)		C-2, 4
4	-	196.18	-	196.07	-		196.96	-
5	12.04 (s; 1H)	164.43	12.01 (s; 1H)	162.93	C-6, 7, 8, 10	12.19 (s; 1H)	158.78	-
6	5.97 (d; *J*=2.4 Hz, 1H)	96.76	6.06 (d; *J*=2.8 Hz, 1H)	95.19	C-4, 7, 9, 10	6.10 (s; 1H)	94.66	C-5, 8, 9, 10
7	5.76 (br s; 1H)	164.59	-	164.19		6.46 (br s; 1H)	154.43	-
7-OMe	-	-	3.82 (s; 3H)	55.79	C-9	-	-	-
8	5.99 (d; *J*=2.4 Hz, 1H)	95.54	6.04 (d; *J*=2.4 Hz, 1H)	94.33	C-6, 9, 10	-	128.38	-
8-OMe	-	-	-	-	-	3.94 (s; 3H)	61.09	C-4, 8
9	-	163.36	-	168.05	-	-	157.50	-
10	-	103.20	-	103.20	-	-	103.19	-
1'	-	130.36	-	147.06	-	-	130.36	-
2'	7.37 (d; *J*=8.8 Hz, 1H)	114.33	7.04 (d; *J*=2,0 Hz, 1H)	112.73	C-2, 1', 4'	6.95 (d; *J*=10 Hz, 1H)	127.81	C-1', 3', 4', 5'
3'	6.95 (d; *J*=8.4 Hz, 1H)	127.85	5.69 (s; 1H)	145.99	-	7.37 (d; *J*=9,2 Hz, 1H)	114.30	C-1', 2', 4', 6'
4'	-	160.14	6.91 (d, *J*=1.6 Hz, 1H)	118.26	C-1'	-	160.13	-
4'-OMe	3.83 (s; 3H)	55.48	-	-	-	3.82 (s; 3H)	55.46	C-4'
5'	6.95 (d; *J*=8.4 Hz, 1H)	127.85	6.88 (d; *J*=8.4 Hz, 1H)	110.72	C-2, 2', 3', 6'	7.37 (d; *J*=9.2 Hz, 1H)	114.30	C-1', 2', 4', 6'
6'	7.37 (d; *J*=8.8 Hz, 1H)	114.33	-	131.59	-	6.95 (d; *J*=10 Hz, 1H)	127.81	C-1', 3', 4', 5'
6'-OMe	-	-	3.92 (s; 3H)	56.14	C-1'	-	-	-

## Data Availability

The data used to support the findings of this study are available from the corresponding author upon request.
